# Physiological and clinical significance of mean circulatory and mean systemic filling pressure

**DOI:** 10.1186/s13613-025-01595-0

**Published:** 2025-11-24

**Authors:** Sheldon Magder, Douglas Slobod, Antoine Vieillard-Baron

**Affiliations:** Department of Critical Care, McGill University Health Centre, 1001 Decarie Blvd, Monrteal, QC H4A 3J1 Canada

**Keywords:** Blood volume, Cardiac output, Compliance, Capacitance, Venous return, Capillary pressure, Right atrial pressure, Venous resistance

## Abstract

A pressure distends blood vessels even when the heart is not beating and there is no blood flow. This is called mean circulatory filling pressure (MCFP). We will first discuss why it is physiologically necessary to have this base pressure. Although all pressures in the vasculature are the same when there is no flow, blood volume distributes based on the compliance of the walls in each compartment. The compliance of systemic venous compartment is by far the largest and contain most of the blood volume. When flow starts, volume redistributes among the vascular regions based on the compliance and resistance draining them. Because of it dominates the total compliance, pressure in the systemic venous compartment changes very little; it is called mean systemic filling pressure (MSFP). Under normal hemodynamic conditions, differences between MCFP and MSFP are trivial because venous compliance is so large compared to all other vascular regions. When cardiac function is maximal, MCFP determines the maximum possible cardiac output. MSFP is significant for two reasons. It is the upstream pressure driving blood back to the right heart. Importantly, it also is the downstream pressure for systemic capillary drainage. Thus, a high MSFP increases the risk of tissue edema. From our review of the studies, the pressure difference from MSFP to the right atrium (RAP) is generally in the 3 to 6 mmHg range so that MSFP can be approximated by adding values in this range to properly measured RAP. Ideally, MSFP should be less than 10 mmHg to limit capillary drainage.

The first mention of a pressure in the vasculature without blood flow is attributed to E.H. Weber in the nineteenth century. This pressure is now called mean circulatory filling pressure (MCFP). Its history is well reviewed by Schipke et al. [1]. E. Starling [2] placed a venous reservoir upstream from his heart-lung preparation to maintain steady filling of his heart-lung preparation in his studies on “the law of the heart” [3]. MCFP was a central part of Arthur Guyton’s analysis of the circulation [4] and also was included in Mathew Levy’s alternative cardio-centric view of the circulation [5]. There recently has been increased interest in the clinical utility of the measurement [6–8]. Before considering this, it is necessary to understand the underlying physiology. Why is it necessary to have a baseline pressure in the circulation when there is no blood flow? Why is MCFP in the range that it normally is? What is the role of MCFP in the determination of cardiac output? What is the difference between MCFP and what is called mean systemic filling pressure (MSFP)? The implications of how MCFP and MSFP are measured are also important. We begin with a discussion on vascular volume and its distribution, then discuss studies that have measured it, and finish with our opinion on its clinical utility.

## Vascular volume and its distribution

**Fig. 1:**
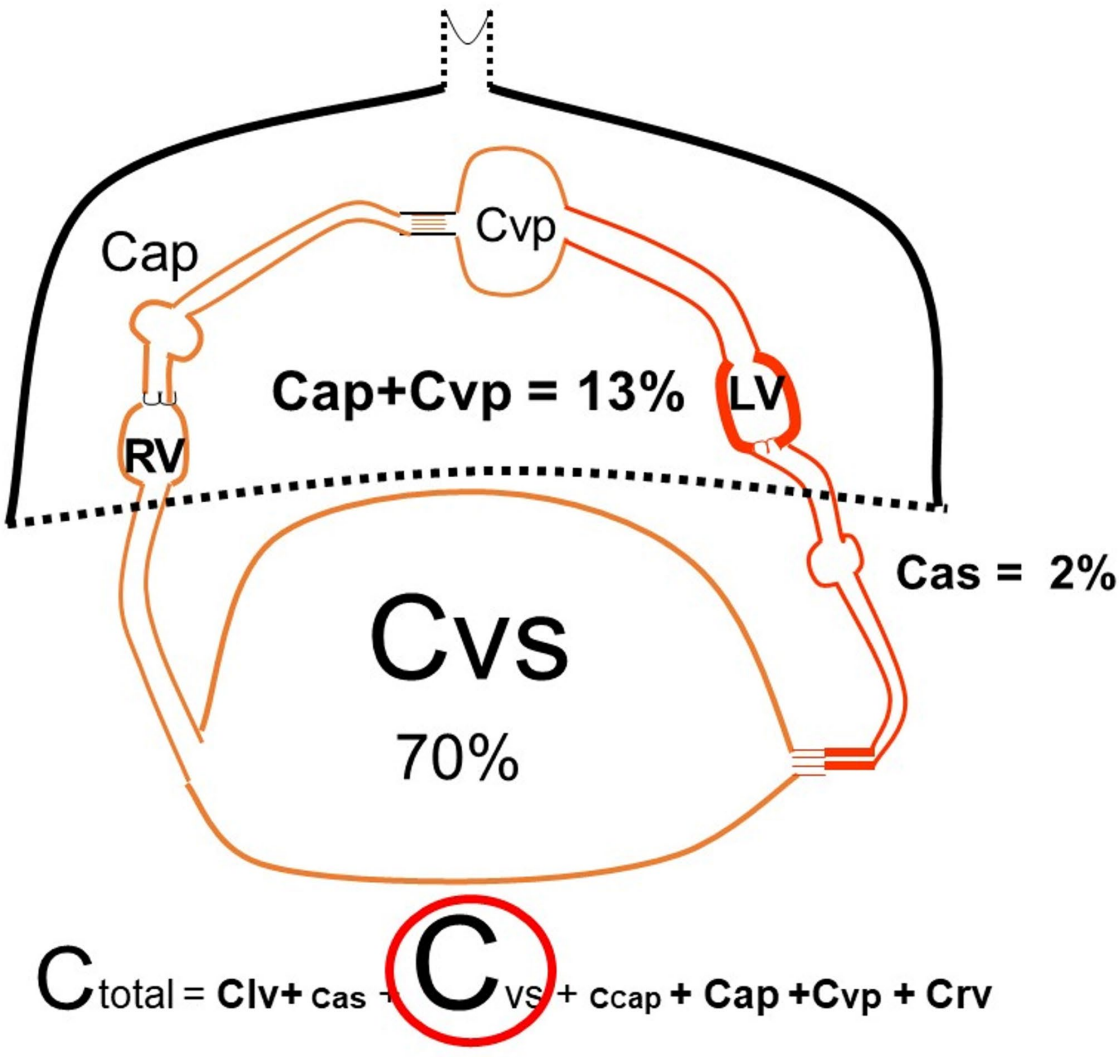
Distribution of vascular compliances C indicates compliance. The largest proportion of vascular volume is in veins and venules (C_v__s_) and dominate total compliance (C_total_). The dark line and dotted line indicate the intrathoracic compartments and the others are the systemic vessels. Percentages are of total vascular compliance. RV = right ventricle, LV = left ventricle, C_as_ = systemic arterial compliance, C_cap_ = capillary compliance, C_ap_ = pulmonary arterial compliance, C_vp_ = pulmonary venous compliance. See text for more details

Typical vascular volume in a 70 kg male is in the range of 5 to 5.5 L, or close to 75 to 80 ml/kg of lean body mass. Approximately 30% of total blood volume is stressed, meaning that it is the volume that stretches vessel walls beyond their resting length [9]. The rest of vascular volume is “unstressed” in that the volume rounds out vessel walls but does not stretch them. Pressure in vessels is determined by the volume they contain and the compliance of the walls. Compliance is the change in volume for a change in pressure. When there is no blood flow, volume distributes throughout the circulation based on the compliance of each region. Total compliance of a system with regions in series as in the circulation is simply the sum of all the compliances of each region in the series (Fig. 1) and accordingly, MCFP is determined by the stressed volume and the sum of all regional compliances [9], including volume in cardiac chambers, pulmonary arteries and veins, and systemic arterial and veins. Systemic veins are by far the most compliant regions and account for 70% of total vascular volume. The compliance of the pulmonary circuit is only 1/7 of that of systemic veins and normally only accounts for 13% of total blood volume 1/7) (Fig. 1) [10]. From an evolutionary point of view this makes sense because it would not be a good to have a large blood volume stored in the lungs! On the systemic side, there also is a large difference between the compliance of arterial and venous vessels. Compliance of veins and venules has been estimated to be between 30 to even 100 times greater than arterial compliance [11]. The value of MCFP is thus dominated by the venous compliance.

**Fig. 2:**
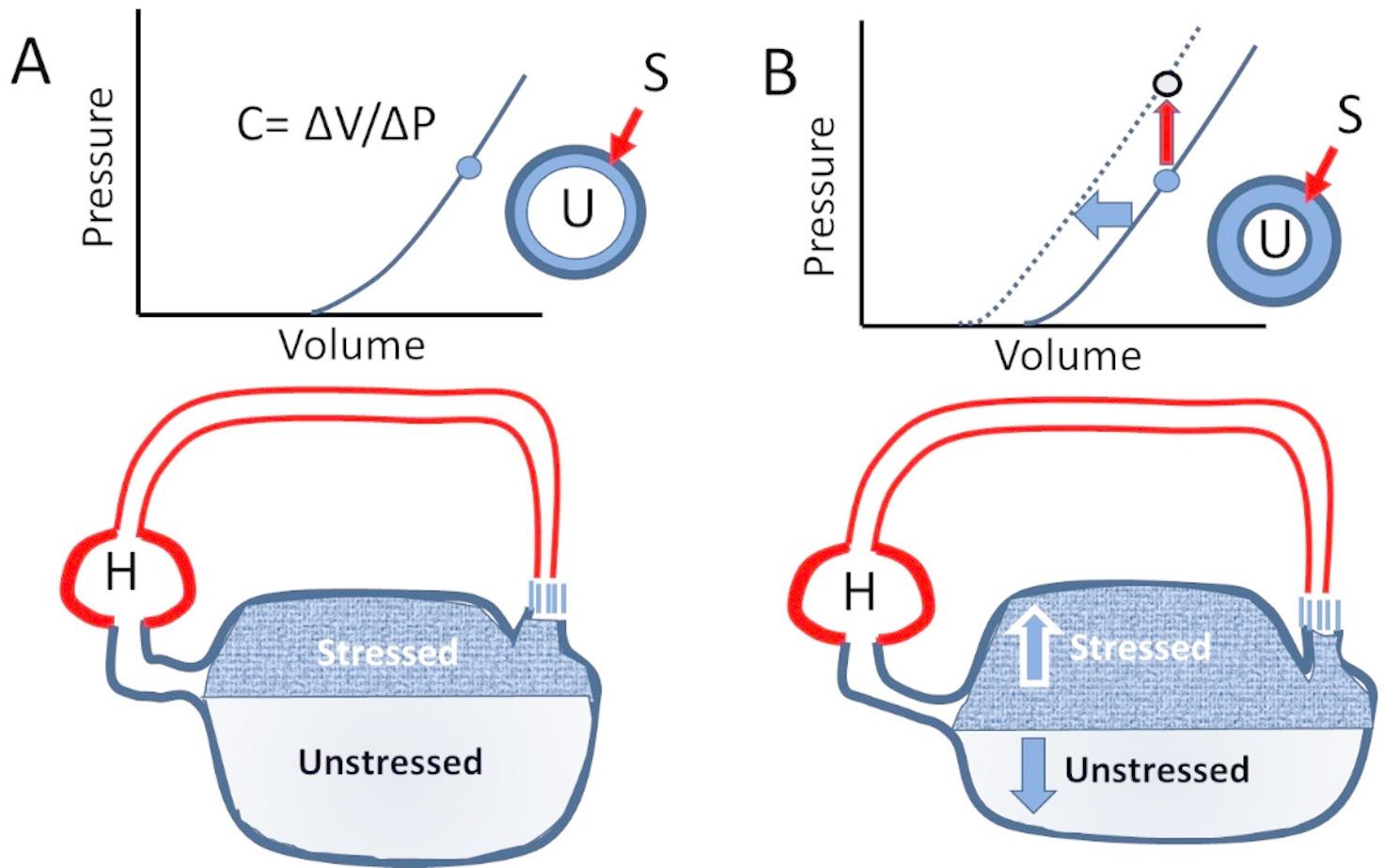
Stressed and unstressed volume with change in capacitance. The bottom figures show simplified examples of circulatory volume. The bulk of blood volume is in the highly compliant venous reservoir. Normally, about 30% of volume is stressed and 70% is unstressed. **A** shows the pressure-volume plot for this reservoir. The slope is change (Δ) in volume (V) for Δ in pressure (P) which gives compliance (C). A cross-section of a vessel also is shown with the unstressed (U) within the white area and stressed volume (S) in blue. **B** shows what happens with a decrease in capacitance. Total volume is unchanged but stressed volume (S) increases and unstressed volume (U) decreases. This shifts the pressure-volume plot to the left so that for the same total volume pressure is increased, but C, the slope, is unchanged

Compliance should not be confused with capacitance. In hydraulic (fluid) systems, the term capacitance includes the stressed volume stretching the compliant vessel walls and unstressed volume that determines the x-intercept of the relationship on a pressure-volume plot (Fig. 2a). The physiological importance of capacitance is that neuro-sympathetic activation can contract vascular smooth muscle and recruit unstressed into stressed volume [12–14].This does not change slope of the pressure-volume relationship, which is compliance, but shifts the line leftward, which and is called a decrease in capacitance. It occurs primarily in the splanchnic bed (Fig. 2b). An often-confusing point is that the equivalent of the vascular compliance term is referred to as capacitance in electric models. Recruitment of unstressed into stressed volume effectively acts an “autotransfusion”. Stressed volume can also rapidly fall if vascular smooth muscle relaxes and vessels dilate. Unstressed volume acts a reserve for the body, but because it creates no pressure it cannot be measured in people.

Drugs alter vascular capacitance but not compliance, which is related to the matrix of the vascular walls. Alpha agonists, vasopressin, angiotensin, and neuropeptide Y all decrease capacitance. Narcotics, vasodilators, and anesthetic agents increase capacitance [13]. Compliance, though, can change over time from chronically increased stress on vascular walls as has been shown in arterial vessels with hypertension [15, 16], and in veins when venous pressures are increased by vascular congestion [17].

### Why is it necessary to have a pressure in the vasculature, i.e. MCFP, when there is no flow?

**Fig. 3:**
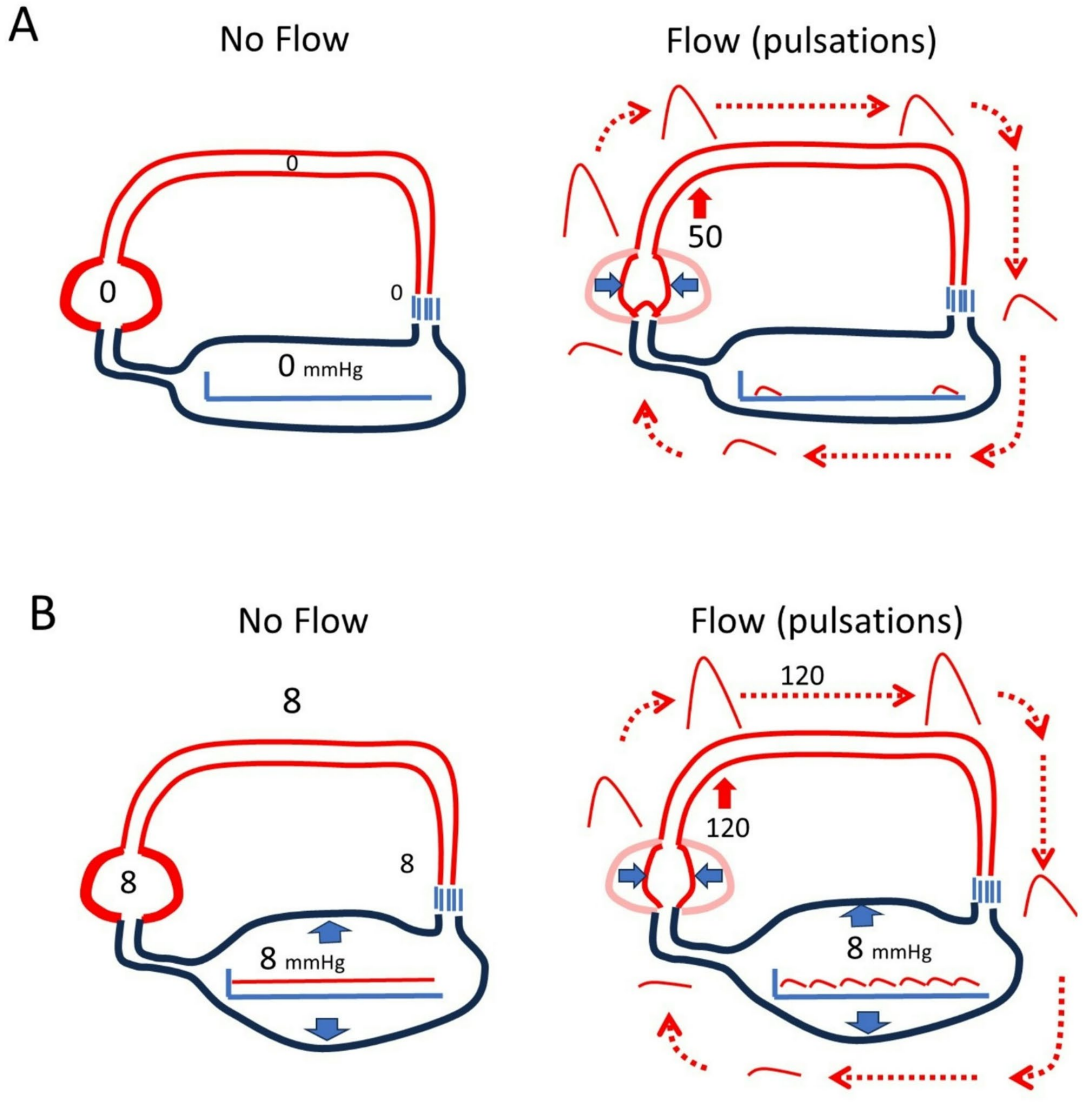
Importance of a baseline pressure for the generation of flow. A Circulatory System with no stressed volume. Right and left ventricles are considered as one unit in this example. Flow only can occur by the heart compressing its unstressed volume. This sends a pulse wave around the system until it returns and refills the heart. The resistance through the system determines the produced pressure, in this case 50 mmHg) and the pressure drop across the circuit during the cycle. Given the time constant based on the product of normal vascular resistance and compliances, only a few beats would be possible per minute. B In this example, baseline pressure is 8 mmHg (~ MCFP) with no flow. Ventricular contraction lowers the diastolic pressure to 4 mmHg and the ventricle refills as it relaxes. Peak arterial pressure is higher (120 mm Hg) and multiple beats can be generated because the heart rapidly fills upon relaxation from the venous reservoirs

MCFP provides a “potential elastic force” that can drive volume out of vessels if the down stream pressure is lowered. For example, even without a beating heart, a cut vein bleeds until MCFP reaches atmospheric pressure [9]. However, unstressed volume does not empty unless drained by gravity. Because the compliance in veins and venules is so large compared to other circulatory regions, systemic veins provide a relatively constant upstream pressure that maintains right ventricular (RV) filling during diastole. The heart works by ejecting blood from the RV during systole and thereby lowering right atrial pressure (RAP) and allowing venous blood to drain back to the RV. If MCFP were zero, stroke volume (SV) could only be generated by compressing unstressed volume in the ventricles (Fig. 3). This volume then would have to be transmitted through all the resistances of the vasculature to get back to the RV for the next contraction. The arterial pulse would be short, peaked, and small unless the cardiac chambers were very large. Preload also could not be altered for that would require a “stressed” volume in the heart, which without valves, would just equilibrate with the venous and arterial compartments. The transit time would be long and heart rate would have to be slow.

### Guyton and the measurement of MCFP

The physiological significance of MCFP was systematically studied by Guyton [18]. He measured MCFP by inducing ventricular fibrillation in dogs and then rapidly pumping blood from the aorta to a jugular vein to equilibrate the two pressures before there was loss of neuro-humeral vascular tone and an increase in capacitance. He identified MCFP as the pressure at which all vascular pressures, including pulmonary vessels, equilibrated. Baseline MCFP in his anesthetized animals was 6.3 ± 0.9 mmHg.

### Measurement of MCFP in humans

Early estimates were made by Starr in deceased patients [19]. Larger and more systematic studies were performed by Repessé et al. [20]. They obtained permission from their ethics committee to measure the equilibrium of venous and arterial pressure in patients who were expected to die imminently. In their first study, the measurements were made one minute after cessation of heart beats and without mechanical ventilation. The average pressure was 12.8 mmHg but was highly variable with a standard deviation of ± 5.6 mmHg. In this large sample of 202 deceased patients, MCFP was not related to gender, severity of illness score, admission diagnosis, fluid balance, or classification of shock. It was moderately higher in those on norepinephrine (14 ± 6 mmHg) and lower in those in whom the decision had been made to forgo life-sustaining therapy. Age was also negatively associated with MCFP. In a second paper, the authors showed that positive end-expiratory pressure (PEEP) and tidal ventilation produced small and equal increases in MCFP and RAP [21]. An increase in MCFP is an important mechanism for the maintenance of cardiac output with application of PEEP [22].

### What is MSFP

The pressure in systemic veins is the upstream pressure for the return of blood to the RV. Because venous volume is so large relative to other regions, it provides a relatively steady pressure source for the return of blood to the RV. As is the case with ejection from the LV, the RV fills with a stroke return. In the steady state, SV and stroke return must be equal. Thus, drainage from systemic veins is matched on each beat by the SV from left ventricle (LV) re-filling the systemic veins. In the steady state, there is very little change in the pressure in systemic veins. This ensures constant RV filling. Because of the importance of the systemic venous pressure during blood flow conditions, it is given it own name, mean systemic filling pressure (M*S*FP compared to M*C*FP ).

Normally, values of MSFP and MCFP are very close. In careful studies in rats, Yamamoto et al. replicated Guyton’s approach by fibrillating the animals and rapidly equilibrating arterial and venous pressures [23]. They then also performed stop-flow studies by occluding the right atrium with a balloon and obtained MSFP at 5 to 10 s after stopping blood flow. In the obstruction studies, arterial pressure remained 10 mmHg above MSFP but there only was a 0.3 mmHg difference between MSFP and MCFP measured with Guyton’s method (MCFP 7.9 ± 0. 9 mmHg versus MSFP 7.6 ± 0.7 mmHg). This gave a ratio of venous to arterial compliance of 60:1. Given this large ratio, it is not surprising that the normal difference between MCFP and MSFP is very small and is easily accounted for by dividing the arterial-venous pressure difference by 1/30 to 1/60.

MSFP has been measured in many animal studies with various techniques including transient right ventricular obstruction [11, 22, 24–26], intravenous infusion of acetylcholine to create transient sinus arrest [27], and transiently stopping a right heart bypass circuit [14, 28, 29]. MSFP in these studies are in the range of 6 to 8 mmHg [30].

MSFP also has been measured in patients undergoing implantation of cardioverter-defibrillator devices for recurrent malignant ventricular arrhythmias. As part of the procedure, ventricular fibrillation is induced to test electrode placement. MSFP is the pressure in central veins 7 to 15 s after the arrest. In Jellinek et al., the average arrest value was 10.2 ± 3.5 and RAP was 7.3 ± 3.1 mmHg before the arrest, indicating a pressure difference for venous return of 3 mmHg. These patients were mechanically ventilated; when positive end-expiratory pressure (PEEP) was increased from 0 to 15 cmH_2_O, RAP rose to 10.0 mmHg and MSFP to 12.7 ± 3.2 so that the MSFP to RAP pressure difference was unchanged. Similar values were observed by Schipke et al.; MSFP was 11.0 ± 5.4 mmHg and RAP 7.5 ± 5.2 mmHg giving a MSFP-CVP pressure difference of 3.5 mmHg. The average difference between arterial and venous pressures during the measurement was 13.2 ± 6.2 mmHg so that the authors concluded that measurement of MSFP requires more than 20 s. However, they failed to appreciate that even a low estimate of the ratio of venous to arterial compliance of 30:1 would only change the values by 0.3 mmHg.

Although values of MSFP and MCFP were close in these studies, this only is true for the mean venous values. Significant regional differences can exist, especially between splanchnic and muscle circulations [14, 28, 29, 31–33]. This is especially evident during exercise when most of the blood flow goes to exercising muscle, which has a much lower venous compliance than the splanchnic circulation [34]. Consequently, the regional equivalent of MSFP in muscle greatly increases the venous return from this region relative to the splanchnic compartment, and is a major factor for the increase cardiac output during exercise [34]. PEEP and ventilation also can alter the distribution of blood flow between splanchnic and non-splanchnic regions and thereby alter regional MSFP as observed by Berger et al. [35].

We used a previously published computational model of the circulation [36] to systematically analyze circulatory factors that alter the relationship of MCFP to average MSFP under potential pathological conditions (Table 1). The model has six compartments: right and left ventricles, pulmonary arterial and venous compartments, and systemic arterial and venous compartments. Because compliances and stressed volume are fixed, MCFP is constant. During steady state flow conditions, volumes and pressures in all compartments can be calculated. Table 1 shows systematic changes in single circulatory parameters with the chosen baseline values. These are what would occur without reflex adjustments. With a stressed volume of 1300 ml, MCFP was 9.3 and the difference between MCFP and MSFP was 0.6 mmHg, close to the normal range. Decreasing left ventricular function, decreasing heart rate, or increasing systemic vascular resistance (SVR), lowered MSFP relative to MCFP. Reduction of left ventricular function to 0% of baseline, reduced MSFP by 4 mmHg from MCFP. This large difference occurred because blood accumulated in the pulmonary and ventricular compartments. An increase in heart rate reduced the difference between MSFP and MCFP below 0.6 mmHg. MSFP was higher than MCFP when SVR was reduced by 50% as would occur in sepsis or exercise [34]. This occurs because systemic venous filling is faster because of the low arterial resistance than venous emptying.

**Fig. 4:**
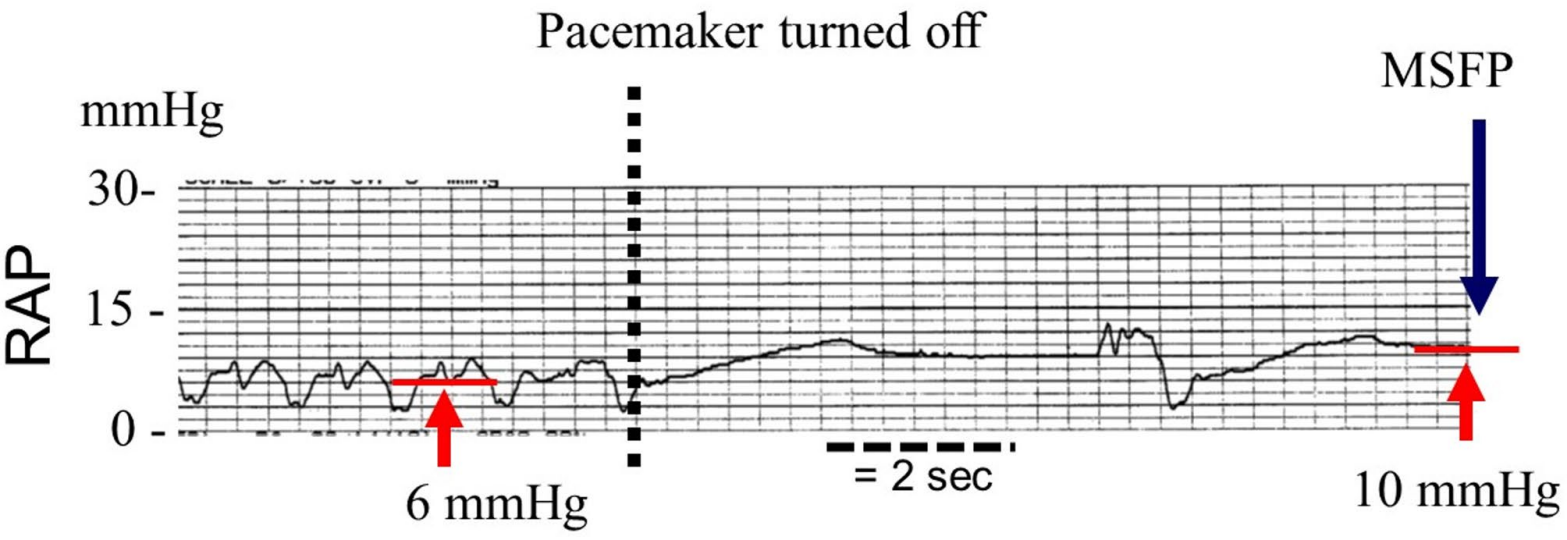
Example of RAP plateau pressure with pacemaker paused Initial RAP is 6 mmHg. At the dashed line the pacemaker was paused. There is an escape beat and non other before the end of the pause. The RAP plateau and thus MSFP was 10 mmHg. The pressure difference for venous return was 4 mmHg. Cardiac output with pacing was 4.4 L/min, heart rate 87 b/min, blood pressure 88/35 mmHg and pulmonary artery pressure 28/14 mmHg. The pressure difference for venous return of only 4 mmHg indicates that small changes in RAP can have large effects on cardiac output

In the course of clinical management, one of the authors (SM) retrospectively collected RAP tracings from 12 patients who died in the ICU and who had orders to not be resuscitated (Table 2). Most were post-cardiac surgery and one had septic shock. Tracings also were obtained from 11 patients who were temporarily paced post-cardiac surgery and pacemakers were paused for 10 to 15 s to determine if there was an escape rhythm. Permission to use the data retrospectively was granted by the director of professional services according to our institutional regulations for retrospective data. The data has not been published previously. An example is shown in Fig. 4. The results were similar to recent studies [1, 21, 37, 38] and allow comparison of measures from patients at death and patients with transient stop flow measurements (Table 2). The standard level of the transducers was 5 cm below the sternal angle and at the level of the stop-cock above the transducer used for zeroing. Reproducibility of measurements in the unit have been previously published [39]. Statistical analysis was not performed because of the small numbers and large non-normal variance, but the means, standard deviations and ranges are listed. In the arrest group, 11 of the 13 were ventilated. Average MCFP was similar to Repesse et al. [20, 37]. As in their studies, variance was large. The final difference between arterial and venous pressure was 3.4 ± 3.9 which is similar to values obtained by Jellinek et al. [38] and Schipke et al. [1] although the differences in two subjects were 8 and 11 mmHg respectively. It is worth noting that in the patient with the largest difference between MSFP and MCFP, the difference between MSFP and pre-arrest RAP still only was 5 mmHg as in most of the other patients.

**Table 1:** Hemodynamic effects of adjustments of single hemodynamic parameters in computerized computational model of the circulation

	MCFP	MSFP	Difference	heart	Vvs	Vas	Vvp	Vap	Q	BP
	mmHg	mmHg	mmHg	ml	ml	ml	ml	ml	L/min	mmHg
Baseline	9.3	8.7	0.6	94	952	97	106	150	5.5	90
Lvves 50%	9.3	7.5	1.8	246	816	82	167	164	4.7	76
Lvves 20%	9.3	5.3	4	214	553	48	391	194	3	49
Rves 20%	9.3	9.1	0.2	101	995	89	75	145	4.7	78
HR 40 b/min	9.3	8.3	1	227	885	41	103	143	3.6	63
HR 100 b/min	9.3	9.1	0.2	72	982	115	63	164	6	98
PVR x5	9.3	8.7	0.6	78	961	94	88	178	5.1	83
SVR 1/2	9.3	9.4	-0.1	43	1031	72	102	150	5.9	52
SVRx2	9.3	7.5	1.8	123	810	136	159	162	4.8	150

**Table 2:** Hemodynamic value 1 minute after death (arrest) or 7-10 seconds after stopping a paced rhythm

	Summary				
		Arrest	Range	Paced	Range
*n*		12		10	
**MSFP**	mmHg	**15.0 ± 3.8**	**11–26**	**14.0 ± 5.5**	**8–27**
RAP	mmHg	12.8*	8–21	9.9 ± 5.0	5–18
Pm - Pra	mmHg	-		4.2 ± 1.6	2–6
RVR	ml/mmHg/sec	-		0.06 ± 0.02	0.02–0.08
CO	L/min	2.8**	1.4–4.9	4.8 ± 1.2	2.5–7.1
Final Part	mmHg	20.4 ± 7.3	14–37	25.2 ± 1.7*	24–31*

MSFP values obtained in paced subjects were very close to the MCFP in the arrest group When adjustments could be made based on assumptions of the final arterial-venous pressure difference, and an estimate of the ratio of arterial to venous compliance, the difference from estimated MCFP was only 0.5 mmHg. The paced data allowed calculation of the pressure difference from MSFP to RAP. This averaged 4 mmHg and was never >6 mmHg. The average venous resistance was 0.06 ml/mmHg/sec, which is the value that has been used in previous modeling studies based on animal studies (Table 2) [14]. These results indicate that small changes in RAP can have a significant impact on the pressure difference for venous return. Three patients in the arrest group and one in the paced group had MSFP greater than 20 mmHg but the MSFP to RAP pressure difference still was ≤ 4 mmHg indicating that the high MSFP/MCFP was due to excessive fluid administration. It is worth noting that although the pressure difference from MSFP to RAP does not change, this does not mean that cardiac output did not. In an animal study, norepinephrine increased MSFP without a change in RAP/CVP or venous resistance because norepinephrine’s beta properties increased cardiac function and cardiac output [40]. A decrease in venous resistance could do the same. Thus, a change in MSFP without knowing what happened to cardiac function or venous resistance is of limited value.

### Which is the more useful value: MCFP or MSFP?

The answer depends on the question asked. MCFP indicates the pressure created by stressed volume and the sum of all vascular compliances when there is no flow. It thus has one value for each set of initial conditions. In contrast, the value of MSFP is dependent on right and left ventricle function and the distribution of vascular resistances at the moment of the measurement. These all can change rapidly even though MCFP and stressed volume are constant (Table 1). However, when cardiac function and systemic vascular resistance are in the normal range, the difference between MCFP and MSFP is very small. We thus argue that MSFP is the more meaningful value and was likely the intercept that Guyton plotted in his graphs of venous return and cardiac function because his plots were during flow conditions and required changes in a right heart bypass flows, an equivalent of cardiac function.

### Why is MSFP normally not higher?

Quantitative considerations of venous pressures help to understand this. The hydrostatic pressure in capillaries is a key determinant of trans capillary filtration. Normal capillary pressure is in the range of 20 to 25 mmHg [41, 42]. MSFP in healthy subjects is likely 8 to10 mmHg or less so that the difference between MSFP and capillary pressure is likely in the 10 to 15 mmHg range. Accordingly, without a significant adjustment in venous resistance, a MSFP of 20 mmHg, would mean that capillary pressure is at least 30 to 35 mmHg, a strong force for filtration. The increase in filtration would be even greater if albumin is decreased since it is the major determinant of the oncotic pressure countering the capillary pressure.

To take it a step further, the pressure gradient for venous return generally is in the 3 to 8 mmHg range so that a RAP of greater than 10 mmHg would require a MSFP in the 13 to 18 mmHg range and thus increased capillary filtration. To protect tissues from congestion, it is important to keep RAP below 10 mmHg when possible. It is not necessary to measure MSFP because it can be estimated simply from the RAP within a small error range. A RAP less than 10 mmHg can be considered to be in a “safe” zone.

### Use of respiratory maneuvers to estimate MSFP

Attempts have been made to measure MSFP by examining changes in RAP and changes in SV during mechanical breaths [7, 43]. These approaches have produced values that are far beyond the physiologically believable range. This is because of the many confounding factors in the measurement. Flow is estimated on the arterial side of the circulation but pressure on the venous side; there is effective “buffering” by the pulmonary compliance that creates a phase shift in the relationship of flow to pressure; there are possible changes in the distribution of flow; and RV function and MSFP change during tidal ventilation and application of PEEP which violates steady state conditions required in Guyton’s analysis of the venous return function. For further discussion see Repessé et al. [21, 37]. The measured values do appear to indicate qualitative changes in MSFP with infusions of catecholamines or fluid loading [43] but these could have been more easily predicted from the change in RAP with the change in cardiac output and knowledge of whether fluids were or were not given. Far more useful clinical information is obtained with changes in RAP and change in cardiac output [44, 45].

### Assessment of MSFP by a mathematical formula

Parkin and co-workers derived a mathematical formula based on arterial pressure, RAP, cardiac output, and anthropomorphic data to derive a value they called mean systemic filling pressure analogue (Pmsf*a*). They successfully used it to guide volume management during hemodialysis [46]. This formula was subsequently included in a commercial monitoring system. Although under simple conditions, such as fluid infusion or removal, this system moves in the appropriate directions, it lacks many variables for most volume issues in the critically ill. These include a lack of accounting for a change in venous resistance, critical closing pressure in the arterial circuit [47], changes in cardiac contractility as shown above, and most importantly right ventricular limitation [48]. Their estimate is likely no better than adding 3 to 6 mmHg to the CVP value.

### Synthesis

MCFP indicates the potential energy of total stressed vascular volume, whereas MSFP is the important clinical value because it determines venous return to the RV. The two measures are usually close but the value of MSFP relative to MCFP can be altered by changes in cardiac function and by the distribution of blood flow because MSFP is a pressure in only one region, albeit the region with the largest compliance and volume. Besides the importance of MSFP being the upstream pressure driving venous return to the heart, it also is the downstream pressure from the capillaries. Thus, high MSFP indicates the potential risk for capillary leak. A RAP greater than 8 to 10 mmHg measured from 5 cmH_2_O below the sternal angle indicates that MSFP is higher than normal and clinicians should be cautious when giving more fluids when RAP is in this range [49, 50]. Higher MSFP can occur without excess vascular volume but by the increased pleural pressure that occurs with mechanical breaths and high positive end-expiratory pressure (PEEP). If more volume is deemed essential for increasing or maintaining cardiac output, this still needs to be done for short periods of time but measures need to be taken to decrease the need for more fluid. In a study in patients with cardiac disease even a RAP greater 6 mmHg was associated with decreased long term kidney function and decreased survival [51]. Because the pressure difference between MSFP and RAP is small, little information is added by a MSFP measurement. The value of MSFP can be reasonably estimated by adding 3 to 6 mmHg to the RAP. An important exception is abdominal compartment syndrome in which flow limitation can occur in the abdomen [52]. We thus suggest making careful RAP measurements and then considering what are the consequences of the estimated value of MSFP for upstream organs such as the liver and kidney [53]. This estimate of MSFP likely is more accurate than proposed measures in the intact circulation. Importantly, once there is limitation of RV filling [48], changes in MSFP and RAP are equal unless there is a change in the resistance to venous return. In that situation, measurement of MSFP gives no additional information beyond that obtained from the RAP measurement and a direct or indirect measure of cardiac output.

## Data Availability

Yes.

## References

[1] Schipke JD, Heusch G, Sanii AP, Gams E, Winter J. Static filling pressure in patients during induced ventricular fibrillation. Am J Physiol Heart CircPhysiol. 2003;285(6):H2510–5. 10.1152/ajpheart.00604.2003 12907428

[2] Patterson SW, Starling EH. On the mechanical factors which determine the output of the ventricles. JPhysiol. 1914;48(5):357–79. 16993262 10.1113/jphysiol.1914.sp001669 PMC1420422

[3] Starling EH. The Linacre lecture of the law of the heart. London: Longmans, Green & Co.; 1918.

[4] Guyton AC, Lindsey AW, Kaufman BN. Effect of mean circulatory filling pressure and other peripheral circulatory factors on cardiac output. Am J Physiol. 1955;180:463–8. 14376522 10.1152/ajplegacy.1955.180.3.463

[5] Levy MN. The cardiac and vascular factors that determine systemic blood flow. Circul Res. 1979;44(6):739–47. 10.1161/01.res.44.6.739 428068

[6] Cecconi M, Aya HD, Geisen M, Ebm C, Fletcher N, Grounds RM, et al. Changes in the mean systemic filling pressure during a fluid challenge in postsurgical intensive care patients. Intensive Care Med. 2013;39(7):1299–305. 23653181 10.1007/s00134-013-2928-6

[7] Maas JJ, Geerts BF, van den Berg PC, Pinsky MR, Jansen JR. Assessment of venous return curve and mean systemic filling pressure in postoperative cardiac surgery patients. Crit Care Med. 2009;37(3):912–8. 19237896 10.1097/CCM.0b013e3181961481

[8] Persichini R, Lai C, Teboul JL, Adda I, Guérin L, Monnet X. Venous return and mean systemic filling pressure: physiology and clinical applications. Crit Care. 2022;26(1):150. 35610620 10.1186/s13054-022-04024-x PMC9128096

[9] Magder S, De Varennes B. Clinical death and the measurement of stressed vascular volume. Crit Care Med. 1998;26:1061–4. 9635656 10.1097/00003246-199806000-00028

[10] Lindsey AW, Banahan BF, Cannon RH, Guyton AC. Pulmonary blood volume of the dog and its changes in acute heart failure. Am J Physiol. 1957;190(1):45–8. 13458406 10.1152/ajplegacy.1957.190.1.45

[11] Samar RE, Coleman TG. Mean circulatory pressure and vascular compliances in the spontaneously hypertensive rat. Am J Physiol. 1979;237(5):H584–9. 495764 10.1152/ajpheart.1979.237.5.H584

[12] Rothe C. Venous system: physiology of the capacitance vessels. In: Shepherd JT, Abboud FM, editors. Handbook of Physiology. The Cardiovascular System. Section 2. III. Bethesda: American Physiological Society; 1983. pp. 397–452.

[13] Rothe CF. Reflex control of veins and vascular capacitance. Physiol Rev. 1983;63(4):1281–95. 6361810 10.1152/physrev.1983.63.4.1281

[14] Deschamps A, Magder S. Baroreflex control of regional capacitance and blood flow distribution with or without alpha adrenergic Blockade. J Appl Physiol. 1992;263:H1755–63. 10.1152/ajpheart.1992.263.6.H1755 1362332

[15] Asmar RG, London GM, O’Rourke ME, Safar ME. Improvement in blood pressure, arterial stiffness and wave reflections with a very-low-dose perindopril/indapamide combination in hypertensive patient: a comparison with Atenolol. Hypertension. 2001;38(4):922–6. 11641310 10.1161/hy1001.095774

[16] O’Rourke MF. The arterial pulse in health and disease. Am Heart J. 1971;82(5):687–702. 4940223 10.1016/0002-8703(71)90340-1

[17] Sorimachi H, Burkhoff D, Verbrugge FH, Omote K, Obokata M, Reddy YNV, et al. Obesity, venous capacitance, and venous compliance in heart failure with preserved ejection fraction. Eur J Heart Fail. 2021;23(10):1648–58. 34053158 10.1002/ejhf.2254

[18] Guyton AC, Polizo D, Armstrong GG. Mean circulatory filling pressure measured immediately after cessation of heart pumping. AmJPhysiol. 1954;179(2):261–7. 10.1152/ajplegacy.1954.179.2.261 13218155

[19] Starr I. Role of the static blood pressure in abnormal increments of venous pressure, 384 especially in heart failure. II. Clinical and experimental studies. Am JM Sc. 1940;40:385.

[20] Repessé X, Charron C, Fink J, Beauchet A, Deleu F, Slama M, et al. Value and determinants of the mean systemic filling pressure in critically ill patients. Am J Physiol Heart Circ Physiol. 2015;309(5):H1003–7. 26209056 10.1152/ajpheart.00413.2015

[21] Repessé X, Vieillard-Baron A, Geri G. Value of measuring esophageal pressure to evaluate heart-lung interactions-applications for invasive hemodynamic monitoring. Annals Translational Med. 2018;6(18):351. 10.21037/atm.2018.05.04 PMC6186557 30370278

[22] Nanas S, Magder S. Adaptations of the peripheral circulation to PEEP. Am Rev Respiratory Dis. 1992;146:688–93. 1519849 10.1164/ajrccm/146.3.688

[23] Yamamoto J, Trippodo NC, Ishise S, Frohlich ED. Total vascular pressure-volume relationship in the conscious rat. Am J Physiol. 1980;238(6):H823–8. 7386641 10.1152/ajpheart.1980.238.6.H823

[24] Samar RE, Coleman TG. Measurement of mean circulatory filling pressure and vascular capacitance in the rat. Am J Physiol. 1978;234(1):H94–100. 623279 10.1152/ajpheart.1978.234.1.H94

[25] Tabrizchi R, Pang CC. Are angiotensin receptors in vascular smooth muscles a homogeneous population? Eur J Pharmacol. 1987;142(3):359–66. 3428350 10.1016/0014-2999(87)90074-4

[26] Abdelrahman A, Tabrizchi R, Pang CC. Effects of beta 1- and beta 2-adrenoceptor stimulation on hemodynamics in the anesthetized rat. J Cardiovasc Pharmacol. 1990;15(5):720–8. 1692931 10.1097/00005344-199005000-00006

[27] Appleton C, Olajos M, Morkin E, Goldman S. Alpha-1 adrenergic control of the venous circulation in intact dogs. JPharmacolExpTher. 1985;233:729–34. 2861278

[28] Deschamps A, Fournier A, Magder S. Influence of neuropeptide Y on regional vascular capacitance in dogs. Am J Physiol. 1994;266:H165–70. 7905715 10.1152/ajpheart.1994.266.1.H165

[29] Deschamps A, Magder S. Effects of heat stress on vascular capacitance. Am J Physiol. 1994;266:H2122–9. 8203611 10.1152/ajpheart.1994.266.5.H2122

[30] Lee RW, Lancaster LD, Gay RG, Paquin M, Goldman S. Use of acetylcholine to measure total vascular pressure-volume relationship in dogs. Am J Physiol. 1988;254:H115–9. 3337249 10.1152/ajpheart.1988.254.1.H115

[31] Green JF. Mechanism of action of isoproterenol on venous return. Am J Physiol. 1977;232(2):H152–6. 842647 10.1152/ajpheart.1977.232.2.H152

[32] Green JF. Pressure-flow and volume-flow relationships of the systemic circulation of the dog. Am J Physiol. 1975;229(3):761–9. 1211468 10.1152/ajplegacy.1975.229.3.761

[33] Mitzner W, Goldberg H. Effects of epinephrine on resistive and compliant properties of the canine vasculature. J Appl Physiol. 1975;39(2):272–80. 1176390 10.1152/jappl.1975.39.2.272

[34] Magder S, Famulari G, Gariepy B. Periodicity, time constants of drainage, and the mechanical determinants of peak cardiac output during exercise. J Appl Physiol (Bethesda Md: 1985). 2019;127(6):1611–9. 10.1152/japplphysiol.00688.2018 31414960

[35] Berger D, Moller PW, Weber A, Bloch A, Bloechlinger S, Haenggi M, et al. Effect of PEEP, blood volume, and inspiratory hold maneuvers on venous return. Am J Physiol Heart Circ Physiol. 2016;311(3):H794–806. 27422991 10.1152/ajpheart.00931.2015

[36] Magder S, Veerassamy S, Bates JH. A further analysis of why pulmonary venous pressure rises after the onset of LV dysfunction. JApplPhysiol. 2009;106(1):81–90. 10.1152/japplphysiol.90618.2008 18845783

[37] Repessé X, Charron C, Geri G, Aubry A, Paternot A, Maizel J, et al. Impact of positive pressure ventilation on mean systemic filling pressure in critically ill patients after death. J Appl Physiol (Bethesda Md: 1985). 2017;122(6):1373–8. 10.1152/japplphysiol.00958.2016 28360123

[38] Jellinek H, Krenn H, Oczenski W, Veit F, Schwarz S, Fitzgerald RD. Influence of positive airway pressure on the pressure gradient for venous return in humans. J ApplPhysiol. 2000;88(3):926–32. 10.1152/jappl.2000.88.3.926 10710387

[39] Magder S, Serri K, Verscheure S, Chauvin R, Goldberg P. Active expiration and the measurement of central venous pressure. J Intensive Care Med. 2016. 10.1177/0885066616678578 27872408

[40] Datta P, Magder S. Hemodynamic response to norepinephrine with and without Inhibition of nitric oxide synthase in Porcine endotoxemia. AmJRespCritCare Med. 1999;160(6):1987–93. 10.1164/ajrccm.160.6.9808019 10588618

[41] Levick JR, Michel CC. Microvascular fluid exchange and the revised starling principle. Cardiovasc Res. 2010;87(2):198–210. 20200043 10.1093/cvr/cvq062

[42] Levick JR. Capillary filtration-absorption balance reconsidered in light of dynamic extravascular factors. Exp Physiol. 1991;76(6):825–57. 1768414 10.1113/expphysiol.1991.sp003549

[43] Persichini R, Silva S, Teboul JL, Jozwiak M, Chemla D, Richard C, et al. Effects of norepinephrine on mean systemic pressure and venous return in human septic shock. Crit Care Med. 2012;40(12):3146–53. 22926333 10.1097/CCM.0b013e318260c6c3

[44] Magder S. An approach to hemodynamic monitoring: Guyton at the beside. Crit Care. 2012;16:236–43. 23106914 10.1186/cc11395 PMC3682240

[45] Magder S. Right atrial pressure in the critically ill: how to measure, what is the value, what are the limitations. Chest. 2016. 10.1016/j.chest.2016.10.026 27815151

[46] Parkin G, Wright C, Bellomo R, Boyce N. Use of a mean systemic filling pressure analogue during the closed-loop control of fluid replacement in continuous hemodiafiltration. J Crit Care. 1994;9(2):124–33. 7920979 10.1016/0883-9441(94)90023-x

[47] Magder S. Starling resistor versus compliance. Which explains the zero-flow pressure of a dynamic arterial pressure-flow relation? Circul Res. 1990;67:209–20. 10.1161/01.res.67.1.209 2364491

[48] Magder S, Slobod D, Assanangkornchai N. Right ventricular limitation: A Tale of two elastances. Am J Respir Crit Care Med. 2022. 10.1164/rccm.202106-1564SO 36257049

[49] Boyd JH, Forbes J, Nakada TA, Walley KR, Russell JA. Fluid resuscitation in septic shock: a positive fluid balance and elevated central venous pressure are associated with increased mortality. Crit Care Med. 2011;39(2):259–65. 20975548 10.1097/CCM.0b013e3181feeb15

[50] Li DK, Wang XT, Liu DW. Association between elevated central venous pressure and outcomes in critically ill patients. Ann Intensive Care. 2017;7(1):83. 28795349 10.1186/s13613-017-0306-1 PMC5549673

[51] Damman K, van Deursen VM, Navis G, Voors AA, van Veldhuisen DJ, Hillege HL. Increased central venous pressure is associated with impaired renal function and mortality in a broad spectrum of patients with cardiovascular disease. J Am Coll Cardiol. 2009;53(7):582–8. 19215832 10.1016/j.jacc.2008.08.080

[52] Magder, S., Physiological complexities of intraabdominal hypertension: taming the elephant. Ann Intensive Care, 2025. 15(1): p. 76. 10.1186/s13613-025-01481-9 PMC12125405 40447876

[53] Pesenti A, Slobod D, Magder S. The forgotten relevance of central venous pressure monitoring. Intensive Care Med. 2023;49(7):868–70. 37294343 10.1007/s00134-023-07101-z

